# The financial impact of participant attrition from randomised trials: a case‐study from the Occupational Therapist Intervention Study (OTIS)

**DOI:** 10.1111/jep.14212

**Published:** 2024-10-22

**Authors:** Athanasios Gkekas, Sarah Ronaldson, Adwoa Parker, David Torgerson

**Affiliations:** ^1^ Department of Health Sciences, York Trials Unit University of York York UK; ^2^ York Health Economics Consortium York UK

**Keywords:** attrition, loss to follow‐up, opportunity costs, retention, RCTs, trial efficiency

## Abstract

**Rationale:**

Loss to follow‐up of participants can compromise the statistical validity of randomised trials. Moreover, it can have financial consequences for trial teams and funders. This study explores the Occupational Therapist Intervention Study (OTIS) where, despite a withdrawal rate of less than 10%, the trial team incurred opportunity costs related to participants who were initially recruited but subsequently decided to withdraw from the trial.

**Aims and Objectives:**

To estimate the cost of participant losses to follow‐up in the OTIS trial and thus introduce a costing framework to research teams on how they could estimate the opportunity costs of participant withdrawal from their randomised trials.

**Methods:**

The participants lost to follow‐up are differentiated by (1) the time point at which they were lost to follow‐up; (2) the treatment group they were allocated to; (3) their response patters to follow‐up questionnaires; these elements were considered to identify the relevant types of attrition. Protocol‐driven costs of trial materials, including administration, print, and shipping, were gathered. We calculated unit costs for each type of attrition by multiplying protocol‐driven and intervention costs with the relevant number of participants. Summing up unit costs by type of loss to follow‐up yields aggregate figures, enabling the estimation of aggregate and average opportunity costs of attrition.

**Results:**

The average cost per participant loss to follow‐up in the OTIS trial is £93.39. The aggregate cost of participant loss to follow‐up is £9,712.45 from the economic perspective of the trial team. Therefore, 1.35% of the allocated funding has been misallocated because of participant loss to follow‐up.

**Conclusion:**

Despite the low attrition rate of the OTIS trial, loss to follow‐up has still generated considerable opportunity costs. It is recommended that decision makers focus on identifying strategies which could improve participant retention in randomised trials to optimise their budget.

## 1 INTRODUCTION

### 1.1 Participant loss to follow‐up in randomised trials

Attrition is one of the key challenges to the statistical validity of randomised controlled trials (RCTs),^1^ which naturally benefit from the feature of randomisation holding other things constant.^2^ Attrition occurs when already recruited participants withdraw from a trial during follow‐up, die during the study or are lost to follow‐up while they receive the intervention they were randomly allocated to.^1^ This phenomenon is capable of introducing selection bias if the dropout rate is differential by treatment group and/or participants’ baseline characteristics, thus introducing uncertainty in a trial's findings and its dissemination.^1^ Thus, attrition may reduce the external validity of the study's results, as the sample size can be skewed so that the final sample differs significantly from the original sample in terms of baseline characteristics.^3^ It is also likely that the patient population be indirectly affected by trial attrition as the treatment they will be receiving after the dissemination of the findings may not necessarily improve their health outcomes as much as other available treatments could, as the treatment effects of proposed (rejected) interventions or treatments may have been wrongly estimated.^4^

Loss to follow‐up in a trial is also expected to have financial consequences for trial teams and research funders. With respect to trial teams, costs can be protocol‐driven and related to the administration and delivery of trial materials to participants lost to follow‐up, as well as related to the data management of such participants. With respect to research commissioners, opportunity costs can arise from foregone resources used for recruiting and retaining participants lost to follow‐up, thus leading to lower return on investment.

### 1.2 The Occupational Therapist Intervention Study (OTIS)

Although it is believed that “*the cost of loss to follow‐up…is very hard to assess*”,^5^ our study introduces a framework for estimating the economic costs of participant losses to follow‐up through a case study from the Occupational Therapist Intervention Study (OTIS); a cohort, pragmatic, two‐arm, open RCT (trial registration number: ISRCTN22202133).^6^ The trial compared the effectiveness of a home environmental assessment and modification tool administered by occupational therapists (OTs) versus usual care for patients at higher risk of falling. With respect to patient recruitment, 19,308 packs were distributed to patients from GP surgeries and previous trial cohorts. 3,100 patients returned their screening questionnaire and an appropriate screening form. The point of informed consent was pre‐screening. Following eligibility checks, falls calendar packs, including falls calendars and a baseline questionnaire, were sent to 1,496 patients, of whom 1,410 returned a baseline questionnaire. 1,331 patients were eventually randomised to the trial. 901 participants were allocated to the usual care group, whereas 430 were allocated to the intervention group. All participants, irrespective of intervention group, received a falls prevention leaflet by their General Practitioner (GP) produced by Age UK at the baseline.^6^

With respect to participant retention the duration of follow‐up was 12 months, consisting of 4‐monthly follow‐up questionnaires sent to the trial participants, along with a return envelope.^6^ All participants received £5 in cash unconditionally along with their final follow‐up questionnaire. 53 out of 430 participants (12.3%) in the intervention group, and 77 out of 901 participants (8.5%) in the control group were lost to follow‐up. Excluding deaths and considering exclusively participants who decided to withdraw from the study, 41 out of 430 participants (9.53%) in the intervention group, and 63 out of 901 participants (7.00%) in the control group were lost to follow‐up; thus, the attrition rate, excluding deaths, is 7.8% (104 out of 1331 randomised participants). There may be significant administration costs, as well as print and shipping costs of trial materials related to the recruitment and the follow‐up of randomised participants lost to follow‐up, acting as resource misallocation to the trial commissioner's budget of £722,096.59 (NIHR Health Technology Assessment Programme 14/49/149).^7^ A CONSORT diagram showing the recruitment and retention of participants in the OTIS trial is available in Supporting Information 1.

### 1.3 Aim

The study aims to estimate the opportunity cost of loss to follow‐up in the OTIS trial by calculating the aggregate and average protocol‐driven costs of participants lost to follow‐up. This estimation will reflect the economic impact of participant attrition on the trial team and the research commissioner. This study also seeks to guide research teams in estimating such costs in their own randomised trials, facilitating more informed budget decisions.

## 2 METHODS

### 2.1 Types of participant loss to follow‐up

There are multiple periods where follow‐up occurred in the OTIS trial after randomisation, at 4, 8, and 12 months through follow‐up questionnaires.^6^ Therefore, attrition in the case study is differential by the following elements:

1) Time point at which a participant was lost to follow‐up (i.e. (a) before receiving the 4‐month follow‐up questionnaire; (b) after having received the 4‐month follow‐up questionnaire but before receiving the 8‐month follow‐up questionnaire; (c) after having received the 4‐ and 8‐month follow‐up questionnaires but before receiving the 12‐month follow‐up questionnaire, and; (d) after having received all follow‐up questionnaires, due to not responding to the 12‐month follow‐up questionnaire.
2) The group a participant was allocated to (i.e. intervention group or usual care group).
3) Whether a participant lost to follow‐up had responded to any of the follow‐up questionnaires they received (i.e., excluding those lost to follow up before receiving the 4‐month follow‐up questionnaire). If someone did not respond to any follow‐up questionnaire within 21 days of receipt, the trial team would incur an additional cost of printing and shipping an extra copy of the questionnaire plus a reminder letter.^6^ These additional costs are the main reason why such a differentiation is needed for estimating the opportunity costs of attrition. If a participant did not formally withdraw from the study or die, the trial team would continue to send them future follow‐up questionnaires.^6^

By considering these three elements, and for each treatment group, we identify four different types of participant loss to follow‐up based on the time point at which loss to follow‐up occurred, with subtypes of attrition related to response patterns to follow‐up questionnaires. Table 1 presents these types plus the number of participants associated with each type. Supporting Information 2 explains the allocation of participants lost to follow‐up in the OTIS trial to each type and subtype of attrition. The reported losses to follow‐up and questionnaire response rates from the published study are considered for determining the sample size by type and subtype of attrition.^6^ The identification of all relevant types and subtypes of attrition was necessary to estimate the costs of loss to follow‐up as accurately as possible, therefore even if no participant belongs to a certain subtype of attrition (i.e., Subtypes 2a, 3a, 3d) we still report the subtype of attrition in Table 1. Note that we define attrition as the decision of a recruited participant to withdraw from a randomised trial. For ethical reasons, losses to follow‐up generated by death events (12 in the intervention group and 14 in the control group) are not considered in the analysis.

**Table 1:** Types of participant loss to follow‐up in the OTIS trial.

Type of loss to follow‐up	Intervention group	Usual care group	All participants
**1. Loss to follow‐up before receiving 4‐month questionnaire**	**16**	**12**	**28**
**2. Loss to follow‐up before receiving 8‐month questionnaire**	**9**	**15**	**24**
2a. Responded to 4‐month questionnaire	0	0	0
2b. Did not respond to 4‐month questionnaire	9	15	24
**3. Loss to follow‐up before receiving 12‐month questionnaire**	**4**	**12**	**16**
3a. Responded to both 4‐month and 8‐month questionnaires	0	0	0
3b. Did not respond to neither 4‐month nor 8‐month questionnaires	2	10	12
3c. Responded to the 4‐month, but not to the 8‐month questionnaire	2	2	4
3d. Responded to the 8‐month, but not to the 4‐month questionnaire	0	0	0
**4. Loss to follow‐up due to not responding to 12‐month questionnaire**	**12**	**24**	**36**
4a. Responded to the 8‐month questionnaire, but not to the 12‐month questionnaire	11	16	27
4b. Responded to neither 8‐month nor 12‐month questionnaires	1	8	9
All types (total)*	**41**	**63**	**104**

### 2.2 Protocol‐driven costs

Having defined attrition in the OTIS trial, the next step is to define the protocol‐driven opportunity costs related to 104 participants who decided to withdraw from the OTIS trial, i.e., economic costs that could have been prevented to occur had loss to follow‐up of these participants from the OTIS trial not occurred, by following the economic perspective of the trial team.

Initially, the trial team incurred costs for recruiting patients to the trial. Given the published trial report, the recruitment packs that all potentially eligible patients received consist of the following case report forms (CRFs): invitation letters, participant information sheets (PIS), consent forms and screening forms.^6^ Evidently, the trial team incurred costs for administering, printing, and shipping recruitment packs to patients, as well as for processing and managing data collected from potentially eligible patients, who were eventually recruited but decided to withdraw from the trial.

Secondly, the trial team incurred costs related to the data management of recruited participants, as well as for administering, printing and shipping falls calendar packs (consisting of monthly falls calendars, the falls prevention leaflet and the baseline questionnaire), reminder letters, and the 4‐, 8‐, and 12‐month follow‐up questionnaires to randomised participants.^6^ Third, it incurred such costs for group newsletters, which were sent to recruited participants at 3 months after randomisation and 2 weeks before receiving their 12‐month follow‐up questionnaire.^6^ Fourth, the trial team incurred financial costs for paying £5 in cash to participants alongside the 12‐month questionnaire. Fifth, shipping costs of monthly falls calendars sent by trial participants were incurred by the trial team; however, data regarding the frequency of participants lost to follow‐up in responding to monthly falls calendars are not available. Information regarding the assumption we made to estimate the number of falls calendars returned by participants lost to follow‐up to the trial team can be found in Supporting Information 3. These costs are not differentiated by treatment group.

There are three main protocol‐driven costing components from the economic perspective of the trial team: administration, print, and shipping costs.

- Administration costs reflect the time required for trial staff to perform administrative tasks related to the recruitment of potentially eligible patients and retention of randomised participants. Relevant tasks for our analysis include recording participant ID numbers, preparing and distributing trial materials, keeping records of questionnaire delivery and data entry for previous timepoints. Under this category fall both the administration costs related to trial materials and the costs associated with data management. The time dedicated to administering trial materials and data for participants lost to follow‐up reflect an opportunity cost, in the sense it could have been allocated to other tasks within the trial if the marginal rates of productivity in other tasks were positive.
- Print costs are direct costs related to printing the relevant material any potentially eligible patient or recruited participant should receive before and after randomisation. Trial materials were usually printed on A4 paper or C5 card in the OTIS trial.
- Shipping costs are direct costs related to sending by post the relevant material any potentially eligible patient or recruited participant should receive before and after randomisation. In the OTIS trial, shipping costs consist of financial costs of pre‐paid freepost envelopes, return freepost postages, print address labels and outgoing postages. Shipping and print costs reflect an opportunity cost, in the sense that trial team could have used these resources for other within‐trial purposes instead.

### 2.3 Administration costs related to participant loss to follow‐up

The administration team in the OTIS trial consisted of a part‐time data clerk (annual salary cost of £10,228 based on 50% full‐time equivalent (FTE)), a part‐time Grade 8 data manager (annual salary cost of £10,795, based on 20% FTE) and a full‐time Grade 5 trial support officer (annual salary cost of £32,033). These salaries were reported, at 2015 price levels, in the grant application of the YTU to the National Institute for Health Research (NIHR), including employer national insurance and workplace pension contributions. Access to the grant application, and thus to staff costs, has been given confidentially by the trial team.

The following formula estimates the aggregate administration cost, considering the data team's salary costs and proportion of time related to administrative tasks relevant for our analysis.

(1) Administrationcosts=Dataclerk′ssalarycosts*υ+Datamanager′ssalarycosts*β+Trialsupportofficer′ssalarycosts*γ

where υ, β, and γ correspond to the proportion of labour time devoted to administration tasks in relation to trial materials. Considering the challenge of estimating these parameters due to differing staff workloads, we make the following assumptions: υ = 100% to reflect the primary focus of data clerks on data entry and processing, *β* = 40% to account for the data management oversight by data managers, and γ = 60% considering the dual role of trial support officers in providing administrative assistance for trial materials (plus keeping records of questionnaire delivery) and supporting trial researchers and investigators. Assuming υ = 100%, we undertake a two‐way sensitivity analysis to explore the impact of varying β and γ jointly between 30% and 100% to evaluate the robustness of our findings given these assumptions. Moreover, since information on task allocation could not be obtained, we assume that the trial staff distributed equally their time in administration tasks across the trial materials.

After solving *Equation* 1, and considering the assumptions made, the total administration costs are estimated to be £33,765.80, with the administration costs per trial material being £4,823.69. To express the administration costs in terms of unit costs, the aforementioned figure is divided by the number of potentially eligible patients or recruited participants to whom the following trial materials were sent: the recruitment pack (*n* = 19,308), the falls calendar pack (*n* = 1496), the 4‐month follow‐up questionnaire (*n* = 1301), the 8‐month follow‐up questionnaire (*n* = 1268), the 12‐month follow‐up questionnaire (*n* = 1237), the first group newsletter (*n* = 1301) and the second group newsletter (*n* = 1237).^6^ Note that the administration costs of monthly falls calendar returns, and reminder letters are assumed to be included already in the unit administration costs of the follow‐up questionnaires, as they formed part of the administrative follow‐up efforts by the trial team.

### 2.4 Print and shipping costs of trial materials related to participant loss to follow‐up

Following correspondence from YTU's member of staff, data about the unit print and shipping costs of the OTIS trial materials have been collected and reported at 2017 price levels. Thus, the administrative unit costs are converted from 2015 price levels to 2017 price levels, using the Consumer price inflation time series data from the World Bank. The 2‐year UK inflation rate from 2015 to 2017 is 3.7%.^8^ The resources determining the unit print and shipping costs, differentiated by trial material are presented in Table 2. Printing costs of trial materials in terms of A4 papers or C5 cards are available, whereas their shipping costs are determined by resources including window and non‐window envelopes, address labels, return and outgoing postage.

**Table 2:** Unit costs related to trial materials.

Resource	Type of Cost	Unit cost	Price Year	Unit	Applicable to							
					Recruitment pack	Falls calendar pack	4‐month follow‐up questionnaire	8‐month follow‐up questionnaire	12‐month follow‐up questionnaire	First group newsletter	Second group newsletter	OT home visit intervention
Administration costs of trial materials, inc. data management	Administration	£4,823.69	2015	Trial material a year	✓	✓	✓	✓	✓	✓	✓	
Non‐ window A4 envelope	Shipping	£8.00	2017	Box of 250 envelopes	✓							
Window A4 envelope	Shipping	£12.49	2017	Box of 250 envelopes		✓	✓	✓	✓			
Pre‐paid A4 freepost envelope	Shipping	£0.19	2017	Envelope	✓	✓	✓	✓	✓			
Printed address label	Shipping	£24.29	2017	Box of 1400 blank labels	✓							
Return freepost postage	Shipping	£0.66	2017	A4 Letter	✓	✓	✓	✓	✓			
Return freepost postage^a^	Shipping (related to monthly falls calendar returns)	£0.44	2017	C5 Letter		✓	✓	✓	✓			
Outgoing postage (A4 size)	Shipping	£0.76	2017	Envelope	✓	✓	✓	✓	✓			
Outgoing postage (A5/C5 size)	Shipping	£0.56	2017	Envelope						✓	✓	
Envelope for newsletters	Shipping	£11.17	2017	Box of 500 envelopes						✓	✓	
Invitation letter	Printing	£0.15	2017	1 A4 page (coloured)	✓							
Participant Information Sheet	Printing	£0.34	2017	4 sides of A4 (coloured)	✓							
Consent Form	Printing	£0.13	2017	1 A4 page (black and white)	✓							
Screening Form	Printing	£0.10	2017	1 A4 page (black and white)	✓							
Contact Form	Printing	£0.10	2017	1 A4 page (black and white)	✓							
Baseline Questionnaire	Printing	£0.32	2017	Booklet (8 pages)		✓						
Falls calendar	Printing	£1.23	2017	Bundle of 18 C5 cards		✓						
Falls Prevention Leaflet	Printing	£3.92	2017	Leaflet (32 pages, coloured)		✓						
Follow‐up questionnaires	Printing	£0.36	2017	Booklet (8 pages)			✓	✓	✓			
Cover Letter	Printing	£0.02	2017	1 A4 page (Black and White)			✓	✓	✓			
Reminder letter^a^	Printing	£0.15	2017	1 A4 page (coloured)			✓	✓	✓			
Newsletter	Printing	£0.25	2017	1 page (coloured)						✓	✓	
OT home visit intervention	Other	£136.53	2017	Participant								✓

^a^ considered separately as trial materials in Table 4

### 2.5 Unit costs related to participant loss to follow‐up

By aggregating the unit administration, print and shipping costs of each trial material *i*, it is possible that their corresponding unit costs be estimated:

(2) Unitcostoftrialmateriali=Unitadministrationcost+Unitprintcost+Unitshippingcost

In addition to the unit costs of trial materials, the trial team incurred a cost for delivering the intervention to participants randomised to the intervention group. This cost consisted of the training the occupational therapists (OTs) received to undertake home visits, the time spent at home visit and on follow‐up telephone call, and the equipment/adaptations installed following the home visit. The figure is provided in the published NIHR report related to the trial, i.e., £136.53.^6^

### 2.6 Average and aggregate costs related to participant loss to follow‐up

The next step is to estimate the unit costs by type of attrition, from the economic perspective of the trial team. To do so, it was crucial to detect which materials participants lost to follow‐up had received, so that the unit costs be estimated accordingly using the unit cost figures. A description of which trial materials participants lost to follow‐up received, by type of attrition, is presented in Table 3.

**Table 3:** Trial materials received by participants lost to follow‐up, by type of attrition.

Type of loss to follow‐up	Intervention group	Usual care group
1. Loss to follow‐up before receiving 4‐month questionnaire due to withdrawal	1x Recruitment pack 1x Falls calendar pack 1x OT home visit 1x First group newsletter 1.56x Falls calendar monthly return	1x Recruitment pack 1x Falls calendar pack 1x First group newsletter 1.56x Falls calendar monthly return
2. Loss to follow‐up before receiving 8‐month questionnaire due to withdrawal		
2a. Responded to 4‐month questionnaire	1x Recruitment pack 1x Falls calendar pack 1× 4‐month questionnaire 1x OT home visit 1x First group newsletter 6.32x Falls calendar monthly return	1x Recruitment pack 1x Falls calendar pack 1× 4‐month questionnaire 1x First group newsletter 6.32x Falls calendar monthly return
2b. Not responded to 4‐month questionnaire	1x Recruitment pack 1x Falls calendar pack 1× 4‐month questionnaire 1× 4‐month questionnaire + reminder letter 1x OT home visit 1x First group newsletter 6.32x Falls calendar monthly return	1x Recruitment pack 1x Falls calendar pack 1× 4‐month questionnaire 1× 4‐month questionnaire + reminder letter 1x First group newsletter 6.32x Falls calendar monthly return
3. Loss to follow‐up before receiving 12‐month questionnaire due to withdrawal		
3a. Responded to both 4‐month and 8‐month questionnaires	1x Recruitment pack 1x Falls calendar pack 1× 4‐month questionnaire 1× 8‐month questionnaire 1x OT home visit 1x First group newsletter 10.51 x Falls calendar monthly return	1x Recruitment pack 1x Falls calendar pack 1× 4‐month questionnaire 1× 8‐month questionnaire 1x First group newsletter 10.51 x Falls calendar monthly return
3b. Not responded to neither 4‐month nor 8‐month questionnaires	1x Recruitment pack 1x Falls calendar pack 1× 4‐month questionnaire 1× 4‐month questionnaire + reminder letter 1× 8‐month questionnaire 1× 8‐month questionnaire + reminder letter 1x OT home visit 1x First group newsletter 10.51 x Falls calendar monthly return	1x Recruitment pack 1x Falls calendar pack 1× 4‐month questionnaire 1× 4‐month questionnaire + reminder letter 1× 8‐month questionnaire 1× 8‐month questionnaire + reminder letter 1x First group newsletter 10.51 x Falls calendar monthly return
3c. Responded to the 4‐month, but not to the 8‐month questionnaire	1x Recruitment pack 1x Falls calendar pack 1× 4‐month questionnaire 1× 8‐month questionnaire 1× 8‐month questionnaire + reminder letter 1x OT home visit 1x First group newsletter 10.51 x Falls calendar monthly return	1x Recruitment pack 1x Falls calendar pack 1× 4‐month questionnaire 1× 8‐month questionnaire 1× 8‐month questionnaire + reminder letter 1x First group newsletter 10.51 x Falls calendar monthly return
3d. Responded to the 8‐month, but not to the 4‐month questionnaire	1x Recruitment pack 1x Falls calendar pack 1× ‐month questionnaire 1× 4‐month questionnaire + reminder letter 1× 8‐month questionnaire 1x OT home visit 1x First group newsletter 10.51 x Falls calendar monthly return	1x Recruitment pack 1x Falls calendar pack 1× 4‐month questionnaire 1× 4‐month questionnaire + reminder letter 1× 8‐month questionnaire 1x First group newsletter 10.51 x Falls calendar monthly return
4. Loss to follow‐up due to not responding to 12‐month questionnaire		
4a. Responded to the 8‐month questionnaire, but not to the 12‐month questionnaire	1x Recruitment pack 1x Falls calendar pack 1× 4‐month questionnaire 1× 8‐month questionnaire 1× 12‐month questionnaire 1× 12‐month questionnaire + reminder letter 1x OT home visit 1x First group newsletter 1x Second group newsletter 1x £5 monetary reward 10.51 x Falls calendar monthly return	1x Recruitment pack 1x Falls calendar pack 1× 4‐month questionnaire 1× 8‐month questionnaire 1× 12‐month questionnaire 1× 12‐month questionnaire + reminder letter 1x First group newsletter 1x Second group newsletter 1x £5 monetary reward 10.51 x Falls calendar monthly return
4b. Responded to neither 8‐month nor 12‐month questionnaires	1x Recruitment pack 1x Falls calendar pack 1× 4‐month questionnaire 1× 8‐month questionnaire 1× 8‐month questionnaire + reminder letter 1× 12‐month questionnaire 1× 12‐month questionnaire + reminder letter 1x OT home visit 1x First group newsletter 1x Second group newsletter 1x £5 monetary reward 10.51 x Falls calendar monthly return	1x Recruitment pack 1x Falls calendar pack 1× 4‐month questionnaire 1× 8‐month questionnaire 1× 8‐month questionnaire + reminder letter 1× 12‐month questionnaire 1× 12‐month questionnaire + reminder letter 1x First group newsletter 1x Second group newsletter 1x £5 monetary reward 10.51 x Falls calendar monthly return

It is assumed that participants who were lost to follow‐up and had responded to a previous follow‐up questionnaire did so without the need for receiving an additional copy of this questionnaire. Combining the information from Table 3 and the unit costs of trial materials and the intervention as presented in Table 2, the unit costs by type of attrition, from the economic perspective of the trial team, are estimated.

The unit cost figures, by type of attrition and intervention group, were multiplied by the number of participants falling into each of these attrition types and intervention groups from Table 1, to estimate the aggregate monetary cost of participants lost to follow‐up to the trial. We assume that all participants from the intervention group lost to follow‐up had received an OT home visit; in the trial's original NIHR report, it is reported that 88.6% of the recruited participants in the intervention group, regardless of attrition status, received the home visit; the intervention cost reported corresponds to estimates using costing data from this subgroup of participants.^6^ The cost per participant lost to follow‐up, from the economic perspective of the trial team, is also estimated.

## 3 RESULTS

### 3.1 Unit costs related to participant loss to follow‐up

By aggregating the administration, print, and shipping unit costs, the unit cost of a recruitment pack is £2.74, the unit cost of a falls calendar pack, including a copy of the baseline questionnaire, falls calendars and the falls prevention leaflet, is £10.47, the unit cost of a 4‐month follow‐up questionnaire is £5.88, the unit cost of an 8‐month follow‐up questionnaire is £5.98, the unit cost of a 12‐month follow‐up questionnaire is £6.08, the unit cost of the first group newsletter is £4.67, the unit cost of the second group newsletter is £4.87, the unit shipping cost of the monthly falls calendar return is £0.44, the unit print cost of reminder letters is £0.15 and the unit cost of the monetary reward is £5.

The NIHR report of the study estimated the total unit cost of the OT home visit intervention to be £136.53 at 2017 price levels,^6^ which is also considered in this study for estimating the costs of attrition to the OTIS trial. The calculations behind the estimation of the administration, print and shipping unit costs are available in Supporting Information 4. All unit costs from the economic perspective of the trial team, by trial material, are summarised in Table 4.

**Table 4:** Unit costs from the economic perspective of the trial team.

Item	Unit administration cost (£, 2017)	Unit print cost (£, 2017)	Unit shipping cost (£, 2017)	Unit cost (£, 2017)
**Recruitment pack**	0.26	0.82	1.66	2.74
**Falls calendar pack**	3.34	5.47	1.66	10.47
**4‐month follow‐up questionnaire**	3.84	0.38	1.66	5.88
**8‐month follow‐up questionnaire**	3.94	0.38	1.66	5.98
**12‐month follow‐up questionnaire**	4.04	0.38	1.66	6.08
**First group newsletter**	3.84	0.25	0.58	4.67
**Second group newsletter**	4.04	0.25	0.58	4.87
**OT home visit intervention**	NA	NA	NA	136.53
**Falls calendar monthly return**	NA^a^	NA^a^	0.44	0.44
**Reminder Letter**	NA^b^	0.15	0.00^b^	0.15
**£5 monetary reward in cash**	NA	NA	NA	5.00

^a^ Unit administration costs for falls calendar monthly returns already considered in the unit administration costs related to the falls calendar pack and the follow‐up questionnaires. Unit print costs related to falls calendars already included in the unit print cost of the falls calendar pack.

^b^ Unit administration costs for reminder letters already considered in the unit administration costs related to the follow‐up questionnaires. Reminder letters were sent alongside an extra copy of a follow‐up questionnaire; hence their additional unit shipping cost is zero.

### 3.2 Average and aggregate costs related to participant loss to follow‐up

The unit costs by type of attrition, from the economic perspective of the trial team, are available in Table 5. The unit cost figures, by type of attrition and intervention group, were multiplied by the number of participants falling into each of these attrition types and intervention groups from Table 1, to generate an aggregate monetary cost of participants lost to follow‐up to the trial team, expressed as the sum of the costing figures included in Table 5. The aggregate, direct costs of participant loss to follow‐up, from the economic perspective of the trial team, are estimated to be £9,712.45. Therefore, the average cost per participant lost to follow‐up, from the economic perspective of the trial team, was:

(3) Averagecostperparticipantlosttofollow‐up=£93.39

**Table 5:** Unit and aggregate costs of attrition from the economic perspective of the trial team.

Type of loss to follow‐up	Intervention group (unit cost; £, 2017 level)	Usual care group (unit cost; £, 2017 level)	Intervention group (aggregate cost; £, 2017 level)	Usual care group (aggregate cost; £, 2017 level)
1. Loss to follow‐up before receiving 4‐month questionnaire due to withdrawal or death	155.10	18.57	2481.66	222.89
2. Loss to follow‐up before receiving 8‐month questionnaire due to withdrawal or death				
2a. Responded to 4‐month questionnaire	163.08	26.55	0	0
2b. Not responded to 4‐month questionnaire	169.12	32.59	1522.06	488.82
3. Loss to follow‐up before receiving 12‐month questionnaire due to withdrawal or death				
3a. Responded to both 4‐month and 8‐month questionnaires	170.91	34.38	0	0
3b. Not responded to neither 4‐month nor 8‐month questionnaires	183.08	46.55	366.16	465.52
3c. Responded to the 4‐month, but not to the 8‐month questionnaire	177.05	40.52	354.09	81.03
3d. Responded to the 8‐month, but not to the 4‐month questionnaire	176.95	40.42	0	0
4. Loss to follow‐up due to not responding to 12‐month questionnaire				
4a. Responded to the 8‐month questionnaire, but not to the 12‐month questionnaire	193.10	56.57	2124.13	905.17
4b. Did not respond to neither 8‐month nor 12‐month questionnaires	199.24	62.71	199.24	501.66

Given that; (1) the estimated cost of attrition is £9,712.45 and; (2) the NIHR awarded £722,096.59 to the trial team,^7^ it means that (£9,712.45/£722,096.59) *100% = 1.35% of the funding has been lost because of participant loss to follow‐up.

The two‐way sensitivity analysis, which jointly varied the proportion of labour time dedicated by the data manager and the trial support officer for administration tasks related to our analysis between 30% and 100%, suggests that the findings remain robust despite the assumptions made on the parameters υ,β and γ, with the aggregate cost of attrition varying between £9,096.50 (when β = γ = 30%) and £10,824.00 (when β = γ = 100%).

## 4 DISCUSSION

### 4.1 Summary of findings and their implications

Despite the low attrition rate in the OTIS trial,^6^ participant loss to follow‐up generated opportunity costs for trial teams and research commissioners.

The average cost per participant lost to follow‐up is estimated to be £93.39. Such a figure includes the administration costs of data management, as well as administration, print, and shipping costs related to trial materials such as the recruitment pack, the falls calendar pack, the follow‐up questionnaires, reminder letters, and newsletters, related to participants lost to follow‐up. Additionally, it includes the cost of the trial's intervention of OT home visit and the final £5 monetary reward. Given the timepoints and frequency of attrition, the aggregate cost is estimated to be £9,712.45. This figure also reflects the economic costs of attrition in the OTIS trial to the trial's funding source, the NIHR,^6^ as £9,712.45 from its allocated funding to the OTIS trial has been lost instead of invested in another clinical study/studies. Finally, since the NIHR allocated £722,096.59 to the OTIS trial,^7^ the aggregate cost of participant loss to follow‐up implies that 1.35% of the budget has been lost.

In 2016/17, the National Institute for Health Research (NIHR) Health Technology Assessment (HTA) Programme allocated approximately £67.6 million to pilot or full‐scale randomised trials.^7^ Assuming other RCTs experienced comparable losses to follow‐up as a percentage of their funding and considering the funding of the NIHR HTA Programme, i.e. 1.35%, the estimated annual cost of attrition for the NIHR would be £908,612. In other words, approximately £910,000 invested from the UK's main health research funder may have not led to improvements in health outcomes for the UK population because of foregone resources originating from participant attrition in its funded studies. Had these foregone resources been allocated optimally, more clinical studies of interest could have secured research funding from the NIHR. In addition, if we consider the £20,000 cost‐effectiveness threshold recommended by the National Institute for Health and Care Excellence (NICE),^9^ which corresponds to a maximum of £20,000 invested per additional Quality‐Adjusted Life Year (QALY) gained, at least 45 QALYs could have been saved had attrition not occurred in NIHR‐funded trials, and these economic resources were invested in cost‐effective health technologies and treatments instead. This figure, however, will understate the opportunity costs for those trials where follow up includes a clinical, face to face, appointment. Therefore, the estimate of the attrition cost to the NIHR HTA programme is probably very conservative.

### 4.2 Strengths and limitations of the study

Aside from the statistical implications of attrition, trial teams face opportunity costs because of administering data related to and providing trial materials for recruited participants lost to follow‐up; this study estimates such costs for a case study which, despite its low attrition rate, faced significant costs due to participant attrition. Our study introduces a costing analysis reflecting these costs. Our approach differentiates the nature of participants lost to follow‐up, according to the time point at which they were lost to follow‐up, the treatment group they were allocated to, and their history of responding to previous follow‐up questionnaires.

Moreover, our findings highlight the importance for trial teams to find methods to reduce participant losses to follow‐up, as the resources foregone could have been allocated more efficiently towards other trial‐related activities. These activities may include the recruitment of additional participants to improve the statistical power of a trial, improving the delivery of interventions, extending the follow‐up period to explore clinical or health outcomes in the longer term, optimising data collection and statistical analysis methods, or implementing a more intensive monitoring of trial‐related activities. This analysis could also be important for trial funders as attrition, which causes resource misallocation, can undermine their efforts to maximise population health outcomes (e.g., a net loss of 45 QALYs if the cost of attrition accounts for 1.35% of the funding allocated by the NIHR to randomised trials) and their return on investment (ROI) within their constrained budget. Finally, trial teams could consider the methodology presented to estimate the economic costs of attrition in their randomised trials, even before commencing the trials, to optimise their budget by considering alternative retention strategies that could reduce loss to follow‐up. Given the opportunity costs of attrition, such a course of action could enhance the research impact of their studies given the budget constraints.

However, the generalisability of our costing methodology may be limited, as the follow‐up data collection methods are not solely limited to postal follow‐up questionnaires. If a trial involves clinical appointments for follow‐up data collection, costs such as staff costs related to follow‐up activities, frequency of clinic visits for follow‐up or reimbursable participant transport costs (e.g., parking fees) to the clinic may also need to be considered in the estimation of the opportunity costs of attrition. Alternatively, if a trial involves telephone interviews, duration of calls, call charges, and trial staff costs related to the conduct of telephone interviews could be relevant opportunity costs. On the other hand, if a trial involves electronic and web‐based questionnaires, costs related to electronic data collection and management may be relevant rather than print and shipping costs of trial materials delivered to participants by post. If a trial involves postal follow‐up methods, the framework presented can be more easily adapted in such a trial. Moreover, if a trial involves alternative interventions, there can be variations in terms of resource utilisation that may require an adjustment to the estimation of the opportunity costs of attrition. For instance, trials of nursing interventions may involve more intensive ongoing training, administrative and monitoring activities, whose costs are incurred by the trial team. On the other hand, trials of medical interventions may involve medical technologies for intervention delivery such as diagnostic tests and more intensive monitoring of adverse events. Thus, trial teams need to adjust the relevant resource items that should be considered for the estimation of the opportunity costs of attrition in their trials according to the follow‐up data collection methods and intervention design. Nevertheless, the framework presented offers a detailed methodology as to how the opportunity costs of attrition were estimated in a case study, the rationale and the approach of which could be applicable to trials of different follow‐up methods or intervention designs.

In addition, we made several assumptions for the estimation of the opportunity cost of attrition in the OTIS trial because of the unavailability of relevant data. For instance, assumptions have been made with regard to the sample size of participants lost to follow‐up corresponding to each type and subtype of attrition in the OTIS trial (see Supporting Information 2), as well as with respect to the number of monthly falls calendars that were returned by post to the trial, by type and subtype of attrition (see Supporting Information 3). It was also difficult to assign precise proportions of labour time devoted to trial‐related administrative activities, something which led to assumptions regarding such proportions; however, our sensitivity analysis has confirmed the robustness of our findings despite the assumptions made.

### 4.3 Direction for future research

The analysis presented could work as a guidance for research teams to estimate the average and aggregate costs of participant attrition in their randomised trials, even before their recruitment phase. In addition, estimates of the aggregate and average cost of attrition could be stated in grant applications to trial commissioners, such as the NIHR, as participant losses to follow‐up affect these institutions due to resource misallocation.

Given the costs of participant attrition in terms of resource misallocation, it seems imperative that trial teams and decision makers identify strategies that could improve participant retention in randomised trials. A study design which could identify such strategies is Studies Within A Trial (SWATs).^10^ Research on SWATs of retention strategies is already in place.^11^ Improving retention in randomised trials could reduce the opportunity costs of attrition and ensure statistical accuracy. So far, no retention strategy has been found to be effective or cost‐effective with high certainty of evidence.^11^, ^12^ Therefore, intensifying research on trial efficiency is important for such strategies to be identified. NIHR has recently recognised the potential impact of SWATs on improving trial efficiency by providing grants of up to £30,000 for eligible SWATs embedded within randomised trials.^13^ In addition to SWATs, individualised approaches, such as altruism, effective communication with participants, flexibility of trial staff, and removing barriers to participation, could be valuable approaches for improving retention, although further evidence is necessary to explore their impact.^14^, ^15^ Finally, we hope that the findings of our study encourage trial teams to understand the wider implications of participant losses to follow‐up in their studies; according to a recent scoping review, only 36.8% of RCT protocols have made reference to plans for promoting participant retention.^16^

## 5 CONCLUSION

Despite the relatively low attrition rate, participant loss to follow‐up in the OTIS trial generated considerable opportunity costs for the trial team, with the total amount approaching £10,000, and for the research commissioner due to the loss of 1.35% of the allocated funding. Thus, we recommend that decision makers focus on identifying strategies which could improve participant retention in randomised trials. The conduct of SWATs for novel and existing retention strategies, as well as the application of individualised approaches to participant retention could positively contribute to the aim of improving trial efficiency, with economic benefits for both trial teams and research commissioners.

## CONFLICT OF INTEREST STATEMENT

The authors declare no conflict of interest.

## Supporting information

Supporting information.

Supporting information.

Supporting information.

Supporting information.

## Data Availability

The data that support the findings of this study are available from the corresponding author upon reasonable request.
