# Supplementary material for: The financial impact of participant attrition from randomised trials: a case‐study from the Occupational Therapist Intervention Study (OTIS)
Source: J Eval Clin Pract. 2024 Oct 22;31(5):e14212. doi: 10.1111/jep.14212 (PMC12239544; doi:10.1111/jep.14212)
Supplement: Supplementary file 1 — Supporting information. [file JEP-31-0-s002.docx]

**Supporting Information 1. CONSORT Diagram of the OTIS trial**

Recruitment packs sent

(n = 19,308)

# **Enrolment**

Excluded (n= 17,977)

- Did not return invitation packs (n = 16,208)
- Not eligible (n =1,511)
- Refused consent (n =68)
- Not sent baseline questionnaire (n=25)
- Did not return baseline questionnaire (n=86)
- Did not return at least one falls calendar within 3 months of being sent the baseline questionnaire (n= 56)
- Withdrawal or death(n=23)

Randomized (n =1,331)

#

Allocated to usual care

(n = 901)

Allocated to intervention

(n= 430)

- Received allocated intervention (n= 381)
- Did not receive allocated intervention (n= 49)
- Withdrew from treatment (n=41)
- Unknown reason (n=7)
- Received visit >12 months after randomization (n=1)

Lost to follow up (n=53)

- Withdrew from the trial (n=41)
- Died (n=12)

Lost to follow up (n= 77)

- Withdrew from the trial (n=63)
- Died (n=14)

**Allocation**

**Follow up**

# **Analysis**

Analysed (n=888)

- Did not return calendar or participant questionnaire (n=13)

Analysed (n=420)

- Did not return calendar or participant questionnaire (n=10)
