# Supplementary material for: The financial impact of participant attrition from randomised trials: a case‐study from the Occupational Therapist Intervention Study (OTIS)
Source: J Eval Clin Pract. 2024 Oct 22;31(5):e14212. doi: 10.1111/jep.14212 (PMC12239544; doi:10.1111/jep.14212)
Supplement: Supplementary file 2 — Supporting information. [file JEP-31-0-s004.docx]

**Supporting Information 2. Types and subtypes of participant loss to follow-up in the OTIS trial**

Figure 3 and Table 5 in the published study of the OTIS trial provide details about rates of participant loss to follow-up and questionnaire response rates [6].

- Before 4-month questionnaire, 16 participants were lost to follow-up in the intervention group, whereas 12 participants were lost to follow-up in the usual care group [6]. It is also reported that 11 participants in the intervention group and 25 participants in the usual care group did not respond to the 4-month follow-up questionnaire [6].
- Before 8-month questionnaire, nine more participants were lost to follow-up in the intervention group and 15 more participants were lost to follow-up in the usual care group [6]. In addition, six participants did not respond to the 8-month follow-up questionnaire in the intervention group and 28 participants in the usual care group did not respond to the 8-month follow-up questionnaire [6].
- Before 12-month questionnaire, four more participants were lost to follow-up in the intervention group and 12 more participants were lost to follow-up in the usual care group [6] . In addition, 12 participants did not respond to the 12-month follow-up questionnaire in the intervention group and 24 participants in the usual care group did not respond to the 12-month follow-up questionnaire [6].

The sample sizes of participants related to all attrition types (i.e. 1,2,3,4) were obtained directly from the aforementioned study findings. Nevertheless, due to data unavailability assumptions about the sample sizes of participants related to the subtypes of attrition (i.e. 2a, 2b, 3a, 3b, 3c, 3d, 4a, 4b) by intervention group were made.

- With regards to Subtypes 2a and 2b, it was assumed that all participants lost to follow-up before the 8-month follow-up period had not responded to the 4-month follow-up questionnaire, if the number of participants not responding to the 4-month questionnaire was not lower than the number of participants lost to follow-up. In the intervention group, this means all nine participants lost to follow-up before the 8-month questionnaire had not responded to the 4-month follow-up questionnaire were assumed to be lost to follow-up (Subtype 2b). Thus, the number of participants lost to follow-up, belonging to Subtype 2a, is zero, in addition to two participants who did not respond to the 4-month questionnaire but were considered not to have been lost to follow-up before the 12-month questionnaire. In the usual care group, since 25 participants did not respond to the 4-month questionnaire and 15 participants were lost to follow-up before the 8-month questionnaire, all participants lost to follow-up before the 8-month questionnaire were assumed not to have responded to the 4-month questionnaire (Subtype 2b), in addition to 10 participants who did not respond to the 4-month questionnaire but were considered not to have been lost to follow-up before the 12-month questionnaire.
- Moreover, in the intervention group, since all participants lost to follow-up before the 8-month questionnaire were assumed not to have responded to the 4-month questionnaire, and since the number of participants not responding to the 4-month questionnaire was greater than the number of participants lost to follow-up by two participants, the cumulative sample size for subgroups 3b and 3d is two. In the control group, the corresponding cumulative sample size for subgroups 3b and 3d is 10, given that 10 participants not responding to the 4-month questionnaire were regarded not to have been lost to follow-up before the 12-month follow-up period.
- Similarly, it was assumed that all participants lost to follow-up before the 12-month follow-up period had not responded to the 8-month follow-up questionnaire. In the intervention group, since four participants were lost to follow-up before the 12-month questionnaire and six participants did not respond to the 8-month questionnaire, the cumulative sample size for subgroups 3b and 3c is four. Also, it seems that two out of six participants who did not respond to the 8-month questionnaire were not lost to follow-up before the 12-month questionnaire; we assume that 50% of such participants, i.e. one participant, also did not respond to the 12-month questionnaire (Subtype 4b). Given that the cumulative sample size for subgroups 3b and 3d is two, it means that two participants fall to subtype 3b and two participants fall to subtype 3c. In the usual care group, since 12 participants were lost to follow-up before the 12-month questionnaire and 28 participants did not respond to the 8-month questionnaire, the cumulative sample size for subtypes 3b and 3c is 12. Also, it seems that 16 out of 28 participants who did not respond to the 8-month questionnaire were not lost to follow-up before the 12-month questionnaire; we assume that 50% of such participants, i.e. eight participants, also did not respond to the 12-month questionnaire (Subtype 4b). Given that the cumulative sample size for subgroups 3b and 3d is 10, it means that 10 participants fall to subtype 3b and two participants fall to Subtype 3c.
- Finally, since 12 participants in the intervention group and 24 participants in the control group were lost to follow-up due to not responding to the 12-month questionnaire, and one participant in the intervention group and 8 participants in the control group fall under Subtype 4b, 11 participants from the intervention group are expected to have been lost from follow-up under Subtype 4a, with the figure in the control group being 16.
