# Supplementary material for: The financial impact of participant attrition from randomised trials: a case‐study from the Occupational Therapist Intervention Study (OTIS)
Source: J Eval Clin Pract. 2024 Oct 22;31(5):e14212. doi: 10.1111/jep.14212 (PMC12239544; doi:10.1111/jep.14212)
Supplement: Supplementary file 3 — Supporting information. [file JEP-31-0-s001.docx]

**Supporting Information 3. Estimation of the monthly falls calendar returns by post by type and subtype of attrition**

Table 4 in the published study of the OTIS trial provide details about rates of monthly falls calendars return rates [6].

Between the baseline and the 4-month follow-up questionnaire, 66 participants did not respond to the monthly falls calendars. Between the baseline and the 8-month follow-up questionnaire, 91 participants did not respond to the monthly falls calendars. Between the baseline and the 12-month follow-up questionnaire, 130 participants did not respond to the monthly falls calendars.

To estimate the number of participants lost to follow-up responding to monthly falls calendars, we assumed that participants not sending a falls calendar in a month x wouldn’t send any subsequent falls calendars. We also assume that such participants had sent all falls calendars related to the previous months prior to the falls calendar related to the month affected. Finally, we assume that participants not sending falls calendars are also lost to follow-up as the calendar return rates were similar to the attrition rates.

Therefore, for the 4-month follow-up period, given that:

1. 26 participants did not send a falls calendar at month 0
2. 12 participants did not send a falls calendar at month 1
3. 6 participants did not send a falls calendar at month 2
4. 9 participants did not send a falls calendar at month 3
5. 13 participants did not send a falls calendar at month 4

Considering the total figure of 66 participants not responding to the monthly falls calendars at month 4, which coincides with the 4-month follow-up questionnaire in terms of time, 39.39% (26 out of 66) of these participants did not respond at month 0, 18.18% (12 out of 66) of these participants did not respond at month 1, 9.09% (6 out of 66 participants) did not respond at month 2, 13.64% (9 out of 66 participants) did not respond at month 3 and 19.70% (13 out of 66 participants) did not respond at month 4.

Therefore, given our assumptions the average rate of monthly falls calendar returns for participants lost to follow-up before the 4-month questionnaire is the weighted average of:

39.39%*0+ 18.18%*1+ 9.09%*2+ 13.64%*3+ 19.70%*4= 1.56 monthly calendar fall returns

Correspondingly, for the 8-month follow-up period, given that:

1. 11 participants did not send a falls calendar at month 5
2. 5 participants did not send a falls calendar at month 6
3. -1 participants did not send a falls calendar at month 7
4. 10 participants did not send a falls calendar at month 8

Considering the total figure of 25 participants not responding to the monthly falls calendars at month 8, which coincides with the 8-month follow-up questionnaire in terms of time, 44% (11 out of 25) of these participants did not respond at month 5, 20% (5 out of 25) of these participants did not respond at month 6, -4% (-1 out of 25 participants) did not respond at month 7 and 40% (10 out of 25 participants) did not respond at month 8.

Therefore, the average rate of monthly falls calendar returns for participants lost to follow-up before the 8-month questionnaire is the weighted average of:

44%*5+ 20%*6- 4%*7+ 40%*8= 6.32 monthly calendar fall returns

Following the same approach, it can be shown that the average rate of monthly falls calendar returns for participants lost to follow-up before the 12-month questionnaire is the weighted average of:

15.38%*9+ 30.77%10+41.03%*11+12.82%*12= 10.51 monthly calendar fall returns
