# Supplementary material for: The financial impact of participant attrition from randomised trials: a case‐study from the Occupational Therapist Intervention Study (OTIS)
Source: J Eval Clin Pract. 2024 Oct 22;31(5):e14212. doi: 10.1111/jep.14212 (PMC12239544; doi:10.1111/jep.14212)
Supplement: Supplementary file 4 — Supporting information. [file JEP-31-0-s003.docx]

**Supporting Information 4. Unit administration, print and shipping costs of trial materials**

**Unit administration costs of trial materials related to participant loss to follow-up**

At 2015 price levels, the unit administration cost of a recruitment pack is £0.25; the unit administration cost of a falls calendar pack, including the baseline questionnaire, is £3.22; the unit administration cost of a 4-month follow-up questionnaire is £3.70; the unit administration cost of an 8-month follow-up questionnaire is £3.80; the unit administration cost of a 12-month follow-up questionnaire is £3.90; the unit administration cost of the first group newsletter is £3.70; and the unit administration cost of the second group newsletter is £3.90. These are the unit administration costs reported at 2015 price levels.

Following the conversion to 2017 price levels [7] , the unit administration cost of a recruitment pack is £0.26; the unit cost of a falls calendar pack, including the baseline questionnaire, is £3.34; the unit administration cost of a 4-month follow-up questionnaire is £3.84; the unit administration cost of an 8-month follow-up questionnaire is £3.94; the unit administration cost of a 12-month follow-up questionnaire is £4.04; the unit administration cost of the first group newsletter is £3.84 ;and the unit administration cost of the second group newsletter is £4.04. The unit administration costs of interest are presented in *Table 4*. The unit administration costs also consider the administration costs related to the monthly falls calendar returns, as they formed part of the follow-up efforts related to data collection from follow-up questionnaires by the trial team and the administration costs of the falls calendar pack.

**Unit print costs of trial materials related to participant loss to follow-up**

The unit print cost of a recruitment pack is £0.82, including the unit print costs of an invitation letter (£0.15), a PIS (£0.34), a consent form (£0.13), a screening form (£0.10), and a contact form (£0.10), all colour printed and sized in A4 papers. The unit print cost of a full falls calendar, consisting of 18 C5 cards, is £1.23. The unit print cost of a baseline questionnaire is £0.32. The unit print cost of the patient falls prevention leaflet is £3.92. The unit print cost of the falls calendar packs, including a copy of the baseline questionnaire and the falls prevention leaflet, is therefore £5.47. The unit print cost of a copy of the 4-, 8-, and 12-month follow-up questionnaires, each of which includes an additional page with a cover letter, is £0.38. The unit print cost of a group newsletter (both first and second) is £0.25. These costs are reported at 2017 price levels and presented in *Table 4.* In addition, the print costs of the reminder letter (1 page-coloured), i.e. £0.15, are considered for participants not responding to follow-up questionnaires. Therefore, these costs are also considered for subtypes of attrition 2b, 3b, 3c, 3d, 4a and 4b.

**Unit shipping costs of trial materials related to participant loss to follow-up**

The unit shipping cost of a recruitment pack is £1.66. The unit shipping costs of a falls calendar pack (including falls calendar plus baseline questionnaire) and a 4-, 8- and 12-month follow-up questionnaire are identical, at £1.66. The unit shipping cost of a group newsletter (both first and second), printed on A5 or C5 paper, is £0.58, which includes the unit cost of an envelope and outgoing postage. These costs are reported at 2017 price levels and presented in *Table 4.* Finally, the shipping costs of the falls calendar monthly returns are considered, with the return freepost postage for a C5 letter costing £0.44 to the trial team.
